# The impact of digital and E-health literacy on college students’ mental health: the chain mediation effect of internet addiction and sleep quality

**DOI:** 10.3389/fpubh.2026.1883365

**Published:** 2026-09-21

**Authors:** Zhen Wang, Xuanyang Lu, Yanshu Ji, Huabin Wu

**Affiliations:** 1 Zhejiang University of Finance and Economics Dongfang College, Haining, China; 2 School of Business, Nanjing University, Nanjing, China

**Keywords:** chain mediation effect, college students, digital health literacy, internet addiction, mental health, sleep quality

## Abstract

There can be no assurance about the mental health of college students in today’s digital information age without their personal digital health literacy. The current academic studies on this mechanism are in their infancy. This paper uses cognitive behavioral theory and stress response theory to build a chain mediation model of internet addiction and sleep quality. The study recruited participants from a pool of 407 college students and provided the students with standard scales, such as the e-HEALS, AMHLAQ, IAT, and ISI-C. The researchers used the SPSS 27.0 software to analyze the data and tested the mediation hypothesis using the Bootstrap method. The findings indicate a positive relationship between digital health literacy and mental health among college students. Additionally, the study found that internet addiction and sleep quality served as mediators. The complete chain was defined as digital health literacy → internet addiction → sleep quality, with the total indirect effect on college mental health measured at 0.6304. The study helped fill an academic void regarding digital health literacy. It demonstrated that internet addiction can be reduced and sleep quality and college students’ mental health can be improved. The study has both academic and professional merit. The study aids colleges in the application of digital intervention strategies and the development of mental health literacy among college students.

## 1 Introduction

With online education as a key element of the ongoing wave of global digital transformation, Internet technology is now an indispensable component of first year university students’ study, life, and health management systems. During the higher education stage, individuals are at the highest level of development of both the body and the mind, actively shaping their social adaptability. Mental health for university students is important, as it impacts both the potential for personal human capital and the sustained positive development of society as a whole. Modern university students are digitally active and interact with a unique environment of fast, fragmented, and continuously changing online information. Although this is helpful, it creates many behavioral problems. Deviant behavioral problems stemming from Internet over-dependence are becoming more visible, as is poor information filtering, the misleading Internet effect. Together, these create a serious, global problem for university students’ mental health (1). Tackling these problems is now a priority for the global higher education system and public health policies (2). From a global perspective, Mental health and digital lifestyles of university students are the focus of public health systems, as they integrate into the health service system for youth (3).

Internationally, the combination of digital technologies and health has been established as the main focus of global public health and educational reforms. The World Health Organization (WHO) continues to assert that improving public digital health literacy is of great strategic importance in raising the health of the population, and has included this in the Global Mental Health Action Plan, directing the public to employ Information Technology (IT) for the management of their health in a more personalized way. The International Association for Higher Education states that digital literacy coupled with mental health interventions in the university curriculum is critical to transforming higher education and creating a psychological counseling service framework responsive to the digital economy (4). Evidence suggests that digital health competencies are critical for managing the mental health of university students and that developing these competencies has the potential to resolve the mental health of young people and improve the overall health of the population (5). Within this context, it is of great relevance to study digital technologies and their impact on the mental health of university students as it pertains to the ongoing higher education and public health reforms occurring as a result of globalization.

According to Norman and Skinner (6), e-health literacy is the fundamental ability of people to use computer-based information and communications technology to solve health-related problems. He further argued that e-health literacy reveals people’s ability to use technology to improve their health management. In the rapid development of information technology, this literacy can determine the users’ capability to effectively utilize the internet resources for the maintenance of their mental health. Numerous researches, both locally and internationally, have shown that the presence of better digital electronic health literacy has brought about more reasoned health-related decision-making along with improvements in one’s mental health (7). The area of public health has shown that higher levels of digital health literacy help reduce the symptoms of mental ill health, especially depression and anxiety, among students, making it one of the most important components of mental health protection for the youth (5). As the Internet continues to improve, the phenomenon of ‘Internet Addiction’ has started to become prevalent, and has manifested itself as one of the new concealed threats to the mental and physical health of the youth.

Certain literature findings focus on issues related to excessive use of virtual spaces leading to maladaptive/anti-social behavior patterns, and other behavioral and psychological problems (8). From the point of view of cost and benefit to Public Health, it can be noted that internet addiction and sleep disorders are positively related, and their interaction negatively affects the mental and physical health of students (9). Sleep is one of the most appreciated indicators of the state of one’s health in the academic community, and it is of particular importance. Various interdisciplinary studies suggest that chronic sleep deprivation is not only the precursor of many psychological problems, but also a warning sign for the diagnosis of a wide range of diseases (10). In this respect, formation of scientific and reasonable sleep habits among students has become one of the priorities of mental health education in higher education institutions (11).

Current research shows that digital electronic health literacy, internet addiction behavior, and sleep quality significantly impact college students’ mental health, yet the research on the chain transmission system between these factors is still developing. Most studies focus on confirming a single mediating effect. Moreover, research on the dynamic interaction and combined effects of internet addiction and sleep quality is still underdeveloped. Given the rapidly evolving digital world, the public health community is beginning to employ a chain multiple mediation model to study the interactions of multi-level complex factors (12). A single mediation model remains the most common method employed by researchers, which limits their ability to truly understand the mechanisms of complex, multi-dimensional variable interaction. In light of increasing mental health challenges within the student community, and the need to improve public health literacy, this study aims to build a chain model through the lens of internet addiction and sleep quality as central mediators. This study will examine the indirect effects of digital electronic health literacy on college students’ mental health through these two pathways. This will provide much needed evidence for the enhancement of college mental health counseling and promote the country’s health knowledge dissemination strategies.

## 2 Research hypotheses and model

### 2.1 Digital e-health literacy and college students’ mental health

The theory of health literacy presented by Simonds provides an important framework for understanding the complex relationship between digital health literacy and the mental health of college students. This theory breaks away from the conventional cognitive restriction of health literacy as knowledge acquisition. Rather, it expands its definition to an individual’s ability to access, critically appraise, make sense of health information and apply it to address the health-related challenges that one may encounter. Research has shown that an individual’s performance in this dimension directly influences the logic of their judgments as well as the methods and means of their decisions, and it provides a strong influence on their health and mental health mechanisms. In the context of the rapid advancement of the information age, the Internet becomes the predominant medium from which contemporary youth access health information, build social connections, and manage their lives. Within this framework, digital electronic health literacy, which is a rapidly developing discipline, is increasingly becoming one of the key factors for strengthening the mental health of youth. Its dominant features are reflected in the ability to harness digital technology to collect, locate, make sense of, and organize data related to mental health, and apply the science of mental health to manage and mitigate complex, interrelated behaviors and mental stress. This new and emerging concept captures not only the demands of modern society, but also the new coping mechanisms of contemporary civilization in response to the various psychological challenges they experience.

Higher education students are pushed to the peak of their physical and mental development. This brings both challenges and opportunities to their self-awareness, relationship management and cognitive processes. In the context of deepening dual transformation (the continual transformation of society and the economy through digitization), the usage of digital resources and the growing and varying quality of Information on the Internet pose new and hard challenges for college mental health management (13). Research shows that students who possess digital-eHealth literacy are able to use scientific and systematic methods to identify authoritative and useful mental health resources and develop their positive psychological cognitive systems; In doing so, they have the ability to resist false and/or harmful psychological resources. They are able to rapidly employ the digital resources of professional psychological services and social support stress systems, and maintain a positive emotional psychological system before and during stress. The quality of their psychological health through the negative emotional reactions of mental stress crises is significantly improved (14). In contrast, students who possess low digital-eHealth literacy are unable to critique the negative emotional sentences of psychological radicalism. They are more vulnerable to negative emotional reactions that of mental stress crises, and develop symptoms of anxiety and/or depression. The above context has led this study to focus on the following important questions:

H1: Digital e-health literacy has a significant positive predictive effect on college students’ mental health.

### 2.2 The mediating role of internet addiction

The Self Determination Theory (SDT) and the Technology Addiction Theory provide a useful combination of theories for analyzing the components of the internet addiction phenomenon. SDT suggests that the real world meets people’s social needs of autonomy, competence and relatedness unevenly, and therefore people often make a choice of compensation in favor of real world over virtual worlds (15). Technology addiction theory complements SDT and argues that the use of technical devices and services to access the Internet and the tendency to create an added value become a problem, depending on control functions of one’s cognition and emotion. Electronic health literacy, as an indication of college students’ assessment of and ease with the use of necessary health-related technology and information, helps to determine the resources used in the online assessment, and also helps establish self-efficacy to make online decisions and may help in decreasing the growth of internet addiction (16). The “Digital Electronic Health Literacy - Internet Addiction - College Students’ Mental Health” model is a typical indirect effect model in which “Internet addiction” is regarded as a major mediating variable. Several studies indicate a significant correlation between this variable and the mental health of college students (17).

Having researched digital e-health literacy, it has been found that those who have a high level of this literacy are more rational, and when cognitive network functions are in play, they can assess their additional attributes and are dependent on the Internet to fulfill their practical needs for information and communication. Such a group tends to be goal-oriented, self-disciplined, and comparatively rational in the face of potential overuse. Through the Digital Platform, they are able to use scientific psychological interventions to manage real-life emotional concerns and thus, decrease the harms of the virtual world and prevent internet addiction. Students who are low in digital electronic health literacy are on the contrary and have a more rigid grasp of the Internet and weaker self-management skills, which causes abuse of Internet resources. Lack of confidence in their emotional self-regulation, a reaction to real-life emotional issues is a more Internet-centered and failure-oriented approach, which can morph into a more unconscious, habitual Internet addiction (18). Internet addiction is becoming a major mental health concern for College Students.

Excessive internet addiction has been associated with declining academic performance, deteriorating social relationships, confusing self-identity, and undermined emotional self-regulation and resilience to discomfort, which in turn increases susceptibility to negative emotions and worsens psychological health (19). Thus, the analysis leads to the following hypothesis:

H2: Internet addiction plays a significant mediating role between digital e-health literacy and college students’ mental health.

### 2.3 The mediating role of sleep quality

Proposed by Hochbaum, Rosenstock, and Kegel in 1952, the Health Belief Model (HBM) was developed based on the association between the mechanisms that form individual health behavior and the structure of thinking and belief of the individual. The main idea of the model is that if people understand the positive and negative consequences of certain behavior on their health, they are more likely to make health-promoting behavior choices. The model also helps analyze the effect of digital health literacy on the mental health of the university students. Digital health literacy is the ability to find, assess, and apply health information in a digital form. Digital health literacy supports individual understanding of the importance of sleep, which can also improve sleep by managing network dependence. A review of the literature has determined that sleep is a necessary condition for the preservation of physical and mental health and is the basis for a positive and healthy mental state. The relationship of “digital health literacy → sleep → mental health of university students” is a significant indirect effect, where sleep is the core mediating variable.

Digital health literacy helps many students recognize the importance of work/rest balance and the use of good sleep hygiene habits, including the reduction of electronic device use before sleep and the optimization of sleep environments. College students with positive sleep health belief systems are more willing to use the Internet less and change their lifestyles to prevent the negative effects of Internet and e-Device overuse on their sleep health. Digital health literacy students are more likely to utilize the sleep-relief-enhancing strategies, including relaxation techniques and progressive improvement of dormitory comfort, habitually and in a way that significantly improves their sleep health (20). Students with low digital health literacy tend to have a low understanding of health-related knowledge and are therefore unable to construct a scientific framework or have positive behavioral expectations. They also have low self-control, and are more likely to engage in Internet use and social networking at the expense of their sleep health. Over the long term, and due to low self-control, students will compromise their sleep health and wellness, and will manifest the symptoms of the class of sleep health disorders known as the insomnias. Jun et al. (21) claim that this deteriorating situation can affect students’ daily and academic performance and cause increased emotional fluctuations, short-term memory decline, and decreased social interaction. As a result, this can cause a vicious cycle of emotional stress and psychological dysfunction that impairs adolescents’ mental health and development. Considering this research background, the present study has developed the following hypotheses:

H3: Sleep quality plays a significant mediating role between digital e-health literacy and college students’ mental health.

### 2.4 Chain mediation between internet addiction and sleep quality

The cognitive-behavioral theory and the stress response theory provide a strong basis for understanding internet addiction and its mediated effects on sleep quality. Cognitive-behavioral theory. States that there is a reciprocal relationship between the cognitive, emotional, and behavioral aspects of a person, and these aspects influence one another (22). As per the stress response theory, individuals are required to cope with challenges by making physiological and psychological adjustments to the external or internal stress factors, and coping with them for a longer time can lead to the creation of additional stressors, therefore, a chain reaction. Digital electronic health literacy, which is an essential skill for students, has raised some misguided beliefs about the use of the internet, leading to an increase in internet addiction (23). As a psychological and social stressor, internet addiction alters the natural physiological processes, and sleep is one of the highly affected. The poor quality of sleep becomes a stressor that further aggravates the psychological problems of individuals, creating a multidimensional relationship. In this sense, the chain mediation effect of internet addiction on the quality of sleep is apparent and is governed by the digital electronic health literacy and mental health of college students.

Overseas scholars have demonstrated a close, significant causal relationship between internet addiction and sleep quality. Numerous studies have shown that excessive internet use disrupts the natural order of circadian rhythm and causes a variety of physiological problems, including sleep disorders and sleep structure disorders (24). Within the university student population, a low level of digital and electronic health literacy demonstrates a low level of understanding of reasonable internet use as well as a low level of self-regulation. Some students use virtual spaces to escape the pressures and emotions of the real world, which contributes to the formation and deepening of internet dependence. There is a vicious cycle of internet addiction, sleep, mental health, and the erosion of an individual’s cognitive control, which not only deepens the mental health crisis, but also contributes to the mental health crisis.

Inadequate sleep hinders brain functions of emotional regulation and cognition and also diminishes one’s ability to manage life’s stressors. For example, sleep deprivation increases anxiety and depression, impairs one’s ability to effectively solve problems, and decreases one’s ability to effectively engage socially. This results in negative consequences for one’s overall mental health (25). Digital electronic health literacy is the framework for individuals’ appropriate understanding and management of online activity. This framework has allowed for the development of the full pathway of “digital electronic health literacy → internet addiction → sleep quality → mental health of college students.” This is the basis of the hypothetical framework for the present study, which allows the researcher to develop a range of research hypotheses and study a range of related issues in the area:

H4: Internet addiction and sleep quality play a significant chain-mediating role between digital health literacy and college students’ mental health.

### 2.5 Research model

Taking into account the existing research assumptions, this study developed a theoretical model with digital electronic health literacy as the primary independent variable. This model conceptualizes internet addiction and sleep quality as significant mediating variables that influence mental health among college students. Figure 1 illustrates the comprehensive framework concerning the influence of digital electronic health literacy on the mental health of college students. To assess the applicability and justification of this theoretical model, the researcher employed SPSS statistical software to conduct a fitting assessment and parameter estimation on the collected data. This study aims to assess, through empirical analysis, the mental health of college students, and offer a foundation to construct mental health programming in a digital context for colleges and universities across the world. In the same vein, this study aims to provide a foundation for the development of mental health programming that incorporates psychological resilience for college students from an international perspective.

**Figure 1:**
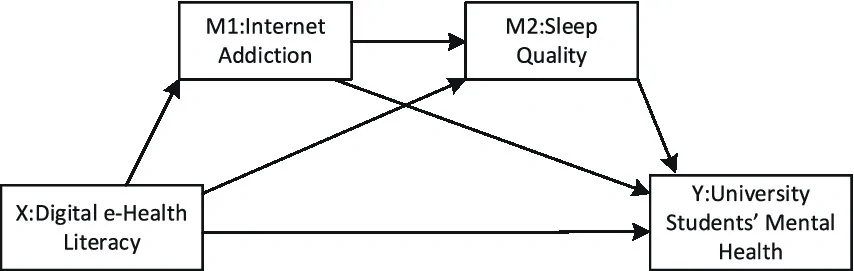
Chained mediating effect model.

## 3 Research methods

### 3.1 Measurement of variables

To ensure data quality, all scales used in this study refer to scales that have been verified in previous research and exhibit good reliability and validity. All scales are measured using a 5-point Likert scoring method, where “1” represents “completely disagree” and “5” represents “completely agree.”

#### 3.1.1 Digital e-health literacy

This study uses the e-Health Literacy Scale (e-HEALS) developed by Norman and Skinner (26). The scale consists of 8 items, covering three dimensions: the ability to access, evaluate, and make decisions about online health information and services. Sample items include “I know how to search for useful health resource information online” and “I know how to use the Internet to answer my own health questions.”

#### 3.1.2 College students’ mental health

This study uses the Adolescent Mental Health Literacy Assessment Questionnaire (AMHLAQ), which was developed by Jorm and its items were validated by Li Danlin et al. (27). The scale consists of 22 items, covering four dimensions: knowledge, recognition, attitude, and behavior. Sample items include “People with depression lose interest and pleasure in most things” and “I can solve the psychological distress I am currently facing.”

#### 3.1.3 Internet addiction

The scale used was the Internet Addiction Test (IAT) developed by Young (28). This scale comprises 20 items, such as “Do you feel that the time you spend online is longer than you intended?” and “Do you neglect your responsibilities because of your internet use?”

#### 3.1.4 Sleep quality

This study uses the Chinese Version of the Insomnia Severity Index (ISI-C) translated by Hong Kong scholars in 2010 (29), which was originally developed by Morin (30). The scale consists of 7 items, such as “difficulty falling asleep” and “difficulty maintaining sleep.” For difficulty-related items, the scoring is “1 = none, 2 = mild, 3 = moderate, 4 = severe, 5 = very severe”; for the evaluation of satisfaction with sleep patterns, the scoring is “1 = very satisfied, 2 = satisfied, 3 = slightly dissatisfied, 4 = dissatisfied, 5 = very dissatisfied.”

### 3.2 Sample data collection

This study adopts a random sampling method, selecting college students from three provinces of Zhejiang, Jiangsu, and Anhui as research participants. Collective testing was conducted through online questionnaire surveys, and a total of 500 questionnaires were collected. After excluding 93 invalid questionnaires with missing answers, regular response patterns and other problems, 407 valid questionnaires were retrieved, with an effective recovery rate of 81.4%. The descriptive statistical information is shown in Table 1. The proportion of male and female respondents is 50.6 and 49.4% respectively, indicating a relatively balanced gender ratio, with slightly more male participants than female; the proportion of participants across the four academic years is relatively average, which are 21.9, 34.6, 23.6, and 19.9%, respectively. This study comprehensively surveyed disciplines including liberal arts, science, medicine, engineering and other fields, covering a wide range of disciplines, among which liberal arts and science account for a larger proportion, reaching 37.8 and 27.8%, respectively. It can be seen that the student sample collected in this study is diverse and highly representative.

**Table 1:** Descriptive statistics of the survey sample.

Basic categories	Indicator	Frequency	Frequency %
Gender	Male	206	50.6%
	Female	201	49.4%
Year	First year	89	21.9%
	Second year	141	34.6%
	Third year	96	23.6%
	Final year	81	19.9%
Subject category	Arts	154	37.8%
	Science	113	27.8%
	Medical	85	20.9%
	Engineering	34	8.4%
	Other	21	5.2%

### 3.3 Data analysis

This study employed SPSS 27.0 statistical package. SPSS was first used to carry out Harman single factor analysis, as well as descriptive and correlational analysis. Thereafter, SPSS with Process macro Model 6 module was employed to examine the chain mediation model, while the Bootstrap method with 5,000 resampling was used to assess the significance of the mediation (31).

This study implemented a mature standardized scale and conducted reliability and validity analysis in order to provide accurate and scientific results. Although all scales used in this study are standardized instruments with established reliability in previous research, we conducted reliability analyses on our sample to ensure that the scales maintained adequate internal consistency in the Chinese college student context, as psychometric properties may vary across different cultural settings and populations. Reporting Cronbach’s *α* for the current sample is a standard practice to enhance the transparency and methodological rigor of the study. Considering the importance of internal consistency in the scale construction process, this article employs Cronbach’s alpha and systematically evaluates the inter-item correlation and overall stability of the items in each dimension. As per statistical standards, Cronbach’s alpha is expressed in the range of values from 0 to 1, and the following criteria are offered for interpretation: when the coefficient is greater than 0.8, it indicates a high level of intrinsic reliability of the scale; A value in the range of 0.7 and 0.8 is said to be slightly inadequate and may be considered as acceptable; Scores less than 0.7 indicate that the questionnaire is in need of revision. Based on data analysis, the values of Cronbach’s alpha for the four key dimensions, “digital health literacy,” “college students’ mental health level,” “internet dependency tendency,” and “sleep quality,” are 0.878, 0.923, 0.918, and 0.909, respectively, which considerably exceed the thresholds, indicating that current research substantially strengthens the theoretical framework.

## 4 Research findings

### 4.1 Test for common method Bias

Because the information in this study comes from respondents’ self-reports, Harman’s univariate analysis was implemented to identify possible common method bias (CMB) concerns. Results from factor extraction without rotation indicated that 10 common factors exhibited cumulative eigenvalues in excess of one. Of these 10 factors, the first common factor explained only 23.229 percent of the total variance, which is well below the acceptable level of the critical threshold (i.e., 40 percent) for multivariate correlation analysis. With this finding, it can be asserted that in the collected data there is no significant overarching main factor, which indirectly corroborates that common method bias in the research is likely to be of negligible concern (Table 2).

**Table 2:** Reliability analysis.

Variable	Cronbach’s *α*	Number of Items
Digital e-health literacy	0.878	8
College students’ mental health	0.923	22
Internet addiction	0.918	20
Sleep quality	0.909	7

This research implemented MPLUS 8.3 to address the concern of common method bias with the inclusion of a modified model. From the original four factor structural model, in each measurement item, independent common method factors that reflect the evaluation tools are embedded. Accordingly, the observed variables are allowed to be related to the underlying dimensions of the theory in the framework of a dual path analysis. Table 3 depicts the changes in model fitting metrics prior to the incorporation of common method factors.

**Table 3:** Model fit following the inclusion of the common method factor.

Model	*χ*^2^	df	*χ*^2^/df	SRMR	CFI	TLI	RMSEA
Four-factor model	190.627	113	1.687	0.030	0.975	0.970	0.041
Four-factor model + common method factor	145.398	102	1.425	0.051	0.986	0.982	0.032
Difference	−45.229	−11	—	+0.021	+0.011	+0.012	−0.009

Incorporating common method factors has clearly shown marked improvement in path model fitting. Based on the results shown in Row 3, the adjusted chi-square statistic shows a rise in chi-square to degree of freedom ratio (CFI) of 0.011, and the Tucker - Lewis index (TLI) of 0.012. The root mean square error of approximation (RMSEA) shows a decrease of 0.009. Despite an increase of 0.021 on the standardized root mean square error (SRMR), it still remains acceptable. The statistical standards have been provided as Δ CFI < 0.1, Δ RMSEA < 0.05. Therefore, the path model has been improved to meet the requirements of structural equation modeling. A thorough analysis reveals that the overall impact of common method bias on the main results of the research is modest, and it has no considerable impact on the analysis of the mediational and moderational effects сохранений and the method’s overall robustness.

### 4.2 Descriptive statistics and correlation analysis

This study examines a number of variables, specifically exploring four key constructs: digital health literacy, college students’ mental health, patterns of internet-related addictive behavior, and internet concerning sleep health. Based on results illustrated in Table 4, it can be concluded that digital health literacy has a statistically significant positive correlation with the mental health of college students (*r* = 0.309, *p* < 0.01) and a statistically significant negative correlation with college students’ internet addiction (*r* = −0.290, *p* < 0.01). Digital health literacy has a statistically significant positive impact on individuals’ sleep (*r* = 0.322, *p* < 0.01). The mental health of individuals has a statistically significant negative impact on internet addiction (*r* = −0.323, *p* < 0.01) and a statistically significant positive impact on individuals’ sleep health (*r* = 0.422, *p* < 0.01). The results of the statistical analysis show that sleep health has a statistically significant negative correlation with internet addiction (*r* = −0.341, *p* < 0.01). Based on the results of the analysis, the variables related to key constructs of the research are significantly correlated at a high level, and form a solid scientific basis for building theoretical constructs and models that will be further substantiated through empirical research.

**Table 4:** Means, standard deviations and correlation coefficients of the study variables (*n* = 407).

Variable	M	SD	1	2	3	4
1. Digital and e-health literacy	32.43	5.46	1			
2. College students’ mental health	82.69	13.39	0.309**	1		
3. Internet addiction	75.73	17.01	−0.290**	−0.323**	1	
4. Sleep quality	26.50	5.27	0.322**	0.422**	−0.341**	1

*p < 0.05; **p < 0.01; ***p < 0.001; the same applies below.

### 4.3 Analysis of the direct and indirect effects of digital e-health literacy on college students’ mental health

Standardized pre-processing of the questionnaire data was performed in this study followed by a multi-level linear regression analysis. The Process macro developed by Preacher and Hayes (Model 6) was used for the multi-level linear regression analysis. To assess the mediating effect, bias corrected percentile self-help approach was applied. The goal is to analyze the effect of digital health literacy on students’ mental health through the lens of internet addiction and sleep quality. Randomly select 5,000 valid research samples, and build and analyze relevant path models.

The following key finding is based on the data depicted in Table 5 and Figure 2. Digital electronic health literacy significantly positively predicts mental health of college students with (*β* = 0.3087, *p* < 0.001). The introduction of the constructs of internet addiction and sleep quality in a multiple regression analysis as moderating variables demonstrated that even when considering the combined effects of several constructs and variables, digital electronic health literacy significantly positively facilitates the enhancement of one’s mental health (*β* = 0.1586, *p* < 0.001). This construct positively, but indirectly, reinforces the protective factor of mental health by decreasing internet addiction (*β* = −0.2901, *p* < 0.001) and by improving sleep quality (*β* = 0.2436, *p* < 0.001). It should be noted that the behavior of internet addiction has a negative effect on sleep quality and the mental health of individuals (*β* = −0.2705, *p* < 0.001); however, improved sleep quality can positively improve mental health of individuals (*β* = 0.3123, *p* < 0.001). All statistical results were verified and showed *p*-values within the significance level of the study.

**Table 5:** Regression analysis of variable relationships in the model.

Regression equation		Overall fit index			Significance of regression coefficients		
Dependent variable	Predictor	*R*	*R*^2^	*F*	*β*	SE	*t*
M1: Internet addiction	X: Digital e-Health literacy	0.2901	0.0841	37.2072	−0.2901	0.0476	−6.0998***
M2: Sleep quality	X: Digital e-Health literacy	0.4131	0.1707	41.5733	0.2436	0.0473	5.1446***
	M1: Internet Addiction				−0.2705	0.0473	−5.7127***
Y: College students’ mental health	X: Digital e-health literacy	0.4856	0.2358	41.4525	0.1586	0.0470	3.3757***
	M1: Internet addiction				−0.1708	0.0473	−3.6099***
	M2: Sleep quality				0.3123	0.0478	6.5311***
Y: College students’ mental health	X: Digital and e-health literacy	0.3087	0.0953	42.6455	0.3087	0.0473	6.5304***

**Figure 2:**
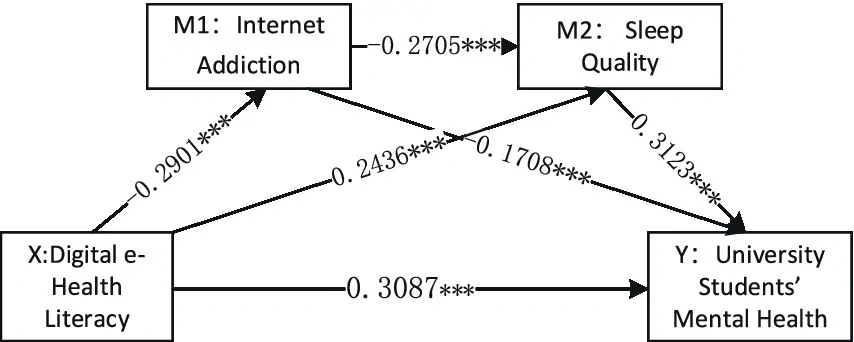
Chain mediation path diagram.

This study uses a mediation analysis framework to examine the roles of internet addiction and sleep quality in the relationship between digital health literacy and mental health of college students. Using empirical data, the analysis (Table 6) shows that the two types of variables act as core moderating components with a cumulative mediation effect value of 0.1501.

**Table 6:** Results of bootstrap mediation analysis.

Effect	Path	Effect	BootSE	BootLLCI	BootULCI	Proportion of effect
Total effect	X → Y	0.3087	0.0473	0.2157	0.4016	100%
Direct effect	X → Y	0.1586	0.0470	0.0662	0.2509	51.38%
Indirect effect 1	X → M1 → Y	0.0495	0.0163	0.0217	0.0852	16.04%
Indirect effect 2	X → M2 → Y	0.0761	0.0242	0.0323	0.1279	24.65%
Indirect effect 3	X → M1 → M2 → Y	0.0245	0.0095	0.0090	0.0462	7.94%
Total indirect effect		0.1501	0.0330	0.0893	0.2186	48.62%

The results of 10,000 simulation inferences using the Bootstrap method suggested a 95% confidence interval of [0.0893, 0.2186]. This range significantly contradicted the null hypothesis and maintained a statistically occupied space of this mediating pathway and contribution to the total effect of about 48.62%. The first chain indirect effect of digital health literacy, internet addiction, and college students’ mental health was estimated at 0.0495. The confidence interval [0.0217, 0.0852] was significantly over zero and represented about 16.04% of the total effect. The second indirect effect of digital health literacy, sleep quality, and college students’ mental health was estimated at 0.0761. The 95% confidence interval [0.0323, 0.1279] was also significantly over zero and represented about 24.65% of the total effect. The indirect effect 3 of the pathway of digital health literacy, internet addiction, and sleep quality, and college students’ mental health was estimated at 0.0245. The confidence interval [0.0090, 0.0462] also was significantly over zero and represented about 7.94% of the total effect. Overall, this brings to light the multi-layered, complex pathways of how multiple mediating factors can influence and modify one’s mental health.

### 4.4 Analysis of differences across academic years

To examine whether there were significant differences among first-year, second-year, third-year, and fourth-year students on the four key study variables (digital health literacy, mental health, internet addiction, and sleep quality), we conducted a one-way Multivariate Analysis of Variance (MANOVA) with academic year as the independent variable. The results showed that the overall multivariate effect was not significant (Wilks’ *Λ* = 0.956, *F* = 1.512, *p* = 0.113), indicating that there were no statistically significant differences across the four academic year groups on the combined dependent variables.

Subsequent univariate ANOVA tests for each variable also revealed no significant differences: digital health literacy [*F*(3, 403) = 1.188, *p* = 0.314], mental health [*F*(3, 403) = 1.429, *p* = 0.234], internet addiction [*F*(3, 403) = 1.036, *p* = 0.377], and sleep quality [*F*(3, 403) = 0.812, *p* = 0.488]. These findings suggest that the relationships observed in our chain mediation model are relatively stable across different academic years, further supporting the robustness of our results.

## 5 Conclusions and discussion

### 5.1 Research conclusions

With digitalization rapidly increasing, and considering the growing psychological health demands of college students, this study aims to develop a theoretical framework based on the health belief model, stress theory, and self-determination theory to analyze the chain mediation effect of digital electronic health literacy, internet addiction, sleep quality, and the psychological health of college students. Data was collected from 407 college students via stratified sampling and a standardized questionnaire. The empirical study utilized correlation analysis, a multiple linear regression model, and the bias correction Bootstrap method. This study aims to investigate the complex mechanisms and the multiple dimensions of the pathway that digital electronic health literacy takes to impact college students’ mental health. The key findings are as follows:

Based on research findings, digital electronic health literacy positively correl with college student mental wellbeing. There is also a strong direct association. Preliminary correlation analysis points to a positive correlation between the two. In estimations that do not account for the possible intervening variables of internet addiction and the quality of sleep, the coefficient is at 0.3087 (*p* < 0.001) suggesting that improvement in digital electronic health literacy positively correl with improvement in mental health. Even in the presence of internet addiction and sleep quality, digital electronic health literacy continues to have a positive and statistically significant (*β* = 0.1586, *p* < 0.001) relationship with mental health, thereby validating H1. This is in alignment with the existing academic literature (7). The current study broadens the existing literature on digital electronic health literacy and mental health by explaining the direct relationship of digital electronic health literacy and mental health, addressing the mechanisms, explaining its value, and suggesting possible applications of this relative the field.

This paper suggests that for college students, as digital technology continues to deepen its integration into their immersion with both learning and experiential scenarios, digital electronic health literacy transcends health literacies and expands to encompass the ability to discern health-related information on the web and engage in health management practices. Learners with a high level of digital electronic health literacy are equipped with specialized competencies that are needed to screen and integrate digital health resources, and therefore have the capacity to develop a comprehensive mental health knowledge framework. Unlike their less digitally literate peers, students with a high level of digital electronic health literacy use supportive and positive digital content to deal with adverse and stressful situations, have the ability to control and alleviate their stressful feelings, and improve their mental fortitude. In contrast, students with a low level of digital electronic health literacy are easily captivated by digital content which focuses on entertainment, and this results in incorrect cognitive structures, cognitive distortions, and emotional instability due to the absence of an effective psychological adjustment system. This correlates with international research that finds that electronic health literacy has a positive impact on the mental health of adolescents. Fostering digital electronic health literacy among college students is crucial for both their mental and physical health and the holistic development of a sustainable community and mental health service framework.

Research has shown that internet addiction is a significant mediator between digital electronic health literacy and mental health of college students. The analyses based on 5,000 bootstrap resampling show that the independent mediating effect of the pathway “digital electronic health literacy → internet addiction → college students’ mental health” is 0.0495, which contributes 16.04% to the total effect, and the 95% confidence interval [0.0217, 0.0852] does not contain zero, which serves to validate the significance of this mediation and fully supports research hypothesis H2. The study findings are consistent with the main perspectives of self-determination and technology addiction theories, that is, the behavior of individuals is not solely determined by their information processing but is linked to self-regulation mechanisms (32). Within this framework, “digital electronic health literacy” encompasses one’s knowledge of the functioning of the internet, as well as one’s ability to control one’s internet use. A high level of digital electronic health literacy helps college students understand the internet as a tool so that they do not use it excessively and become addicted to it. These students tend to use digital resources to learn emotional management, thus decreasing their dependence on the virtual world to fulfill their needs for achievement and belonging.

The use of the internet can have various detrimental effects on a student’s mental health. They can suffer academically, have poor social skills, and have social adaptation disorders, as well as social anxiety. Immersing oneself in a digital space can lead to poor self-regulation, poor time management, and negative emotions of anxiety, depression, and loneliness when faced with life stressors (33). This explains that while developing students’ digital and electronic health literacy, higher education should focus on developing students’ healthy internet usage habits.

Studies have indicated that quality of sleep shows a substantial degree of mediation between digital electronic health literacy and mental health of college students. Based on 5,000 bootstrap resampling analyses, the independent mediating effect value of “digital electronic health literacy → sleep quality → college students’ mental health” is 0.0761, which consumes a 24.65% basis, and the 95% confidence interval [0.0323, 0.1279] does not cover zero, suggesting the mediation pathway is statistically significant, and thus fully endorses the research hypothesis H3. This finding aligns with the central theory of the health belief model: the individual’s health behavior is shaped by health cognition and value systems (34). Students with enhanced digital electronic health literacy can obtain organized knowledge of sleep hygiene from reliable network sources, can recognize the significance of maintaining regular sleep and the need to reduce nighttime use of digital devices to improve the quality of sleep, and are aware of the potential physical and mental health consequences that can be caused by chronic sleep disorders. In view of these, students are likely to follow a regulated pattern of sleep and adopt a healthy lifestyle, which enhances their quality of sleep.

Being a critical link in the body’s physiological control, good-quality sleep is the foundation of mental health, and it also improves the stress and the psychological resilience of the young students. In addition to this, it can reduce the cognitive decline and emotional instability that can be caused due to the build-up of fatigue (35). However, the lack of some essential knowledge in Digital Electronic Health is likely to have students develop one-sided or even wrong sleep beliefs, modify their lifestyle caused by too much online entertainment and/or the overexposure to information, and consequently reduce the efficiency of their sleep. In addition to having a negative effect on the brain, and on the cognitive and emotional regulation function, long-lasting sleep disturbance damages health in a cycle of “deterioration of health—increased negativity—damage to mental health” (36). The studies discussed above establish that sleep quality is a key variable that can link Digital Electronic Health Literacy and Mental Health, and they provide evidence that is important for the construction and improvement of Mental Health education in universities.

Fourthly, internet addiction, through chain mediation, affects how digital electronic health literacy is related to mental health among university students. The results of the Bootstrap method based test show that the value of chain mediation for the pathway of digital electronic health literacy leading to internet addiction and then to sleep quality and, finally, to mental health of university students, is equal to 0.0245, which is 7.94% of the total effect in the 95% confidence interval, [0.0090, 0.0462]. Since the interval does not contain zero, the effect of the chain mediation is confirmed, and thus, H4 is verified. Of this effect, the total indirect effect of internet addiction and sleep quality is 0.1501, which is 48.62% of the overall effect, meaning that digital electronic health literacy is related to the mental health of university students mainly through the indirect effects of internet addiction and sleep quality. This chain mediation can be explored using cognitive behavioral theory and the theory of stress response. According to cognitive behavioral theory, an individual coping with a problem involves an integrated cognitive and emotional process. The malfunction of this process may lead to inappropriate behaviors and increased psychological burden. Stress response theory indicates that external stressors lead to a series of psychological and physiological responses. Among these, a particular response may become a stressor itself, leading to a chain reaction.

This study shows that the absence of digital electronic health literacy has resulted in some college students misconstruing the functional aspects of Internet tools. This has caused students to depend on the Internet and develop Internet addiction. As a permanent behavioral stressor, Internet addiction can initiate hypophasic disorders and other ailments of a hypophasic state through various ways. The overuse of electronic devices can disturb the circadian cycle and cause difficulty in sleeping (37). The deterioration of the quality of sleep has also become a hypophasic stressor, and created a bidirectional negative feedback loop. The first of which is the Internet addiction → decline in sleep quality → decline of self-control → Internet addiction. The sleep deprivation also affects the emotional regulation of the brain and the cognitive functions of the brain and subsequently causes emotional disorders such as anxiety and depression when faced with the stress of everyday life. This leads to a decrease in the individuals problem-solving and social adaptation skills and causes a negative and lasting effect on the mental health of college students (38). The research results on the chain mediation effect outlined above have surpassed the boundaries of traditional single mediation research and illustrated the complexity and multidimensional nature of the issues of college students’ mental health.

Ideal outcomes are difficult to achieve with individual intervention measures. This finding gives educators a way to better design and manage strategies to preserve and enhance college students’ mental wellbeing in a digital environment. In practice, universities should devote energy to improving students’ digital and electronic health literacy, along with special counseling programs for internet addiction and improvement of sleep quality, in a comprehensive and multi-faceted way to ensure the psychological health of college students.

### 5.2 Theoretical contributions

Based on the global public health demand for promoting college students’ mental health in the digital era and empirical data from a Chinese college student sample, this study systematically reveals the multiple pathways and chain transmission mechanism through which digital e-health literacy affects mental health. It achieves theoretical expansion and deepening at three levels: extending health literacy theory, constructing an interdisciplinary integration framework, and supplementing local public health evidence, providing new theoretical support for public health research on youth mental health in the digital era.

Firstly, it has expanded the application boundaries of health literacy theory within the field of youth mental health public health, clarifying the theoretical value of digital and e-health literacy as a core personal health resource. The core concept of health literacy theory has evolved from basic health information literacy to encompass an individual’s comprehensive ability to access, understand, evaluate and apply health information (39); however, existing research has largely focused on its role in physical health and chronic disease management, with insufficient exploration of its mechanisms of action regarding youth mental health in digital contexts (40). This study defines digital e-health literacy as a key public health resource for college students to maintain mental health in the digital age. It not only validates its direct positive impact on mental health but also reveals multi-level indirect transmission pathways, significantly extending the application of health literacy theory to the field of youth mental public health. Furthermore, this study combines the requirements of the resource gain principle of Resource Theory, this study elucidates how digital e-health literacy can foster a virtuous cycle of “high health literacy → healthy behaviours → psychological resource gain → improved mental health” by regulating health behaviours and reducing the depletion of psychological resources. This achieves a cross-theoretical integration of health literacy theory and Resource Theory, providing a novel theoretical perspective for research on youth mental health resources in the public health sector (41).

Secondly, this study integrates multidisciplinary theories to construct a comprehensive mechanism analysis framework from the public health perspective, which makes up for the theoretical defects of single-mediator research. Existing studies on digital e-health literacy and mental health are mostly limited to direct association verification or single mediator analysis, failing to fully explain the temporal correlation and synergistic effect among variables, and lack a systematic theoretical interpretation framework. This study integrates the Health Belief Model, cognitive behavioral theory, self-determination theory and stress response theory to construct an integrated model including direct effect, dual independent mediating effects and chain mediating effect, which completely presents the whole chain transmission process of digital e-health literacy from the cognitive level→behavioral level→physiological level→psychological level. This framework breaks through the limitations of traditional single mediator analysis, clearly reveals the internal logic of the synergistic effect of multiple variables, provides a standardized and replicable theoretical analysis paradigm for analyzing the complex relationship among digital health behaviors, sleep health and mental health in the public health field, and also offers theoretical reference for subsequent research on the mechanism of youth digital health in public health.

Finally, this study supplements local public health empirical evidence for the interdisciplinary field of digital health and mental health, and provides theoretical support from the Chinese context for international research. Current research on digital e-health literacy is mostly originated from Western developed countries, and its conclusions are based on Western digital environments and youth group characteristics, which cannot be fully adapted to China’s local public health context (42). Chinese college students are exposed to a highly penetrated digital living environment and face unique academic and employment pressures, so the association between their digital health behaviors and mental health has local particularity (43). Based on a sample of Chinese college students, this study verifies the mechanism of digital e-health literacy’s effect on mental health, and clarifies that information screening and online behavior self-control are the core dimensions through which digital e-health literacy affects mental health in the local context (44). This study not only echoes the conclusions of international public health research, but also supplements empirical evidence from non-Western contexts, promotes cross-cultural theoretical dialogue in the field of digital health and mental health, and enriches the theoretical system of global youth mental health public health research.

### 5.3 Implications for practice

The empirical conclusions of this study closely align with the needs of public health intervention for college students’ mental health and digital health governance, providing a implementable, full-chain practice path that conforms to public health specifications for universities, families, and society to collaboratively promote youth mental health, and can directly serve the formulation of campus health policies and the improvement of digital health literacy.

Firstly, a systematic framework for cultivating digital health literacy should be established to strengthen the foundations of youth mental health public health. Digital health literacy serves as a core public health lever for preventing psychological risks and interrupting the transmission of unhealthy behaviours; universities should integrate it into comprehensive student education and campus public health service systems (45). Universities may introduce compulsory courses and specialized seminars on digital health literacy to systematically teach skills in retrieving online health information, distinguishing between fact and fiction, assessing risks, and applying knowledge scientifically. Particular emphasis should be placed on cultivating students’ ability to discern information related to mental and sleep health, thereby helping them establish a scientifically grounded framework for digital health literacy (46); Establish authoritative campus health information service platforms to integrate high-quality digital health resources, providing students with convenient and reliable channels for mental and sleep health support, thereby mitigating the public health risks posed by false health information (47); simultaneously, implement a home-school collaborative cultivation model, devising tiered intervention plans tailored to students with varying levels of digital health literacy, to facilitate the transformation of digital health literacy from mere awareness into sustainable health management behaviours.

Secondly, implement precision public health intervention for internet addiction to cut off the key risk transmission path. Internet addiction is an important risk factor for college students’ mental health in the digital age, and also the starting point of the chain transmission mechanism. Universities should include the prevention and control of internet addiction in the scope of campus public health monitoring and intervention. Through online civilization education, theme class meetings, peer presentations and other forms, guide students to establish a tool-rational concept of internet use, and reduce purposeless and excessive internet use behaviors; establish a monitoring mechanism for campus internet use behaviors and an early warning system for psychological risks, and combine mental health screening data to early identify students with internet addiction tendency and implement early intervention (37); enrich offline campus public cultural and social practice activities, provide students with multiple channels for real social interaction and self-realization, and reduce emotional dependence on the virtual internet; for students who have already developed internet addiction, link mental health centers and families to carry out collaborative intervention, and build a closed-loop full-process public health service of “screening-assessment-intervention-follow-up.”

Thirdly, promote standardized public health management of sleep health to strengthen the physiological foundations of mental wellbeing. Sleep quality serves as a critical link between digital health behaviours and mental health, and is a core intervention target for campus public health (48). Higher education institutions should utilize campus social media, thematic education and public lectures on sleep science to disseminate scientific knowledge about sleep and promote sleep hygiene guidelines, thereby helping students develop sound beliefs regarding sleep health (49); guide students to adopt healthy behaviours such as avoiding electronic devices before bed, maintaining regular sleep schedules and engaging in pre-sleep relaxation, to minimise the disruption of sleep rhythms caused by digital device use (50); optimise the sleep environment in halls of residence, strengthen the management of campus schedules, and create a quiet, comfortable and regular rest environment to provide the physical infrastructure for sleep health (51); provide professional psychological counseling and sleep intervention guidance for students with sleep disorders, and where necessary, collaborate with medical institutions to provide diagnostic and treatment services, fully integrating sleep health management into the public health service system for college students’ mental health.

Build an integrated home-school-community collaborative public health intervention network to improve the effectiveness of mental health promotion. The multi-path impacts of digital health literacy on mental health determine that single-subject intervention can hardly achieve optimal outcomes, and it is necessary to construct a collaborative public health governance system of “school–family–society.” Colleges and universities should strengthen the construction of teaching staff for mental health education and digital health service teams, improve the service functions of psychological counseling centers, and enhance the professional level of campus mental health public health services (52); establish a normalized home-school communication mechanism to share real-time information on students’ internet use, sleep status and mental state, and form a joint force for home-school co-education (53); collaborate with social professional mental health service institutions and public health departments to expand off-campus health support resources, build a full-cycle youth mental health public health security network covering screening, intervention, rehabilitation and follow-up, and promote the development of college students’ mental health education in a more scientific, standardized and inclusive direction.

### 5.4 Research limitations and outlook

This study still has certain limitations, and can be deepened and improved from multiple aspects in the future. This study adopts a cross-sectional questionnaire survey design and only obtains static data at a specific time point, which cannot strictly verify the causal relationship and long-term dynamic effects between variables, and is difficult to present the possible two-way causal interaction pattern of internet addiction and sleep quality; the research sample only covers 407 college students from some regions of China, with limited coverage of regions and university types, insufficient sample representativeness, and the generalizability of the conclusions needs to be improved; this study only sets internet addiction and sleep quality as chain mediating variables, does not unpack the multi-dimensional differentiated effects of digital health literacy, nor does it include moderating variables at the individual, family and school levels, so the discussion on the mechanism of action is not comprehensive enough; in addition, data are only collected through self-report method, which leads to certain subjective bias. In the future, longitudinal tracking design and controlled experiments can be used to verify the causal relationship between variables, and the scope of national and even international samples can be expanded to improve the external validity of the conclusions; the dimensions of digital health literacy can be decomposed, and mediating variables such as coping style and self-efficacy as well as moderating variables such as growth mindset and family support can be introduced to construct a moderated mediation model; meanwhile, objective internet use and sleep data and mixed research methods can be combined to reduce subjective bias, further improve the research model, and provide more accurate empirical evidence for public health intervention on college students’ mental health.

## Data Availability

The original contributions presented in the study are included in the article/supplementary material, further inquiries can be directed to the corresponding author.
